# Topical Digitoxigenin for Wound Healing: A Feasibility Study

**DOI:** 10.3390/bioengineering5010021

**Published:** 2018-03-05

**Authors:** Xinchi Feng, Cuifen Wang, Yunhui Xu, Joel Turley, Zijian Xie, Sandrine V. Pierre, Jinsong Hao

**Affiliations:** 1 Department of Pharmaceutical Science and Research, School of Pharmacy, Marshall University, One John Marshall Drive, Huntington, WV 25755, USA; xiaochi0211@163.com (X.F.); wangc@marshall.edu (C.W.); turley37@live.marshall.edu (J.T.); 2 School of Chinese Materia Medica, Tianjin University of Traditional Chinese Medicine, Tianjin 300193, China; 3 Marshall Institute for Interdisciplinary Research, Marshall University, Huntington, WV 25703, USA; xuy@marshall.edu (Y.X.); xiez@marshall.edu (Z.X.); pierres@marshall.edu (S.V.P.)

**Keywords:** digitoxigenin, wound healing, full-thickness excision wound, human dermal fibroblast, alginate

## Abstract

(1) Background: Cardiotonic steroids have been found to stimulate collagen synthesis and might be potential wound healing therapeutics. The objective of this study was to evaluate the feasibility of digitoxigenin and its topical formulation for wound healing; (2) Methods: In the in vitro study, the human dermal fibroblast cells were treated with digitoxigenin and collagen synthesis was assessed. In the in vivo study, digitoxigenin was applied to excisional full-thickness wounds in rats immediately after wounding and remained for three days, and wound open was evaluated over 10 days. A digitoxigenin formulation for topical administration was prepared, and the in vitro release and in vivo wound healing effect were investigated; (3) Results: The expression of procollagen in human dermal fibroblast was significantly increased with the exposure to 0.1 nM digitoxigenin. Topical application of digitoxigenin in olive oil or alginate solution for three days significantly decreased the wound open in rats. Similarly, topical administration of the developed digitoxigenin formulation for three days also significantly increased wound healing. No wound healing effects were observed at days 7 and 10 after wounding when digitoxigenin was not applied; and, (4) Conclusions: It was possible to deliver digitoxigenin using the developed formulation. However, the wound healing effect of digitoxigenin and its mechanisms need to be further investigated in future studies.

## 1. Introduction

Chronic wounds are one of the major clinical challenges in the world. It is estimated that the annual cost on the treatment of chronic wounds is over $25 billion [1]. Due to aging population and increased number of people with diabetes and obesity worldwide, the financial burden of treating chronic wounds is increasing. Current treatment options of chronic wounds are less effective. Thus, there is an urgent need for the development of novel wound healing therapeutics. Herbal medicines have been used over 5000 years and their enduring popularity may be attributed to the perception that they cause minimal unwanted side effects. Discovering novel therapeutics from herbal medicines remains a promising approach for drug development [2].

Cardiotonic steroids (CTSs) are a class of natural products that are commonly found in many herbal plants. CTSs have been used for the treatment of cardiac diseases since Hippocrates and the mechanisms of action are associated with Na/K-ATPase [3]. Recently, researchers found that a signal cascade involving EGFR and PLC could be initiated by CTSs via their binding to Na/K-ATPase, resulting in an increase in collagen expression [4,5,6]. These findings suggest that CTSs may be potential wound healing therapeutics by stimulating collagen synthesis, which is one of the most important mechanisms in wound healing [7]. A previous study revealed that digoxin significantly promoted wound healing by stimulating collagen synthesis and could be a potential wound healing therapeutics [4]. After application of digoxin in olive oil on the excisional wounds in rats, wound closure was significantly accelerated and a greater amount of dermal collagen was detected in the wound area as compared with the vehicle control group. This study also demonstrated that olive oil was necessary for the wound healing effect of digoxin.

CTSs can be divided into two distinct groups: cardiac glycosides (e.g., digoxin, ouabain, and digitoxin) and cardiac aglycones (e.g., digoxingenin, ouabagenin, and digitoxigenin). Digitoxigenin is a cardiac aglycone and it has more favorable physicochemical properties for drug delivery than digoxin, such as smaller molecular weight and greater lipophilicity. Particularly, our pervious in vitro Na/K-ATPase assay study showed that digitoxigenin inhibited the Na/K-ATPase with an inhibitory minimum concentration (IC_50_) of 0.22 μM, which was lower than the IC_50_ for digoxin of 1.76 μM (data not shown). This implies that digitoxigenin showed more affinity with Na/K-ATPase and may be a better candidate for wound healing than digoxin. Despite the stimulated collagen synthesis and wound healing effect of digoxin [4], it is not known if digitoxigenin exhibits the similar wound healing effect as cardiac glycosides, like digoxin. Therefore, it is necessary to prove that digitoxigenin can stimulate collagen synthesis and accelerate the wound closure. 

Olive oil was found as a carrier necessary for the delivery of digoxin due to its lipophilicity [4]. As digitoxigenin is relatively hydrophobic with a measured olive oil/water partition coefficient of 17.75 [8], olive oil is expected to be an important carrier for the wound healing effect of digitoxigenin. However, the drug in olive oil solution is inconvenient for topical application. Therefore, we developed a topical formulation capable of loading both digitoxigenin and olive oil. Alginate has been used in wound healing due to its unique gelling property in contact with polyvalent cations, such as calcium [9]. Calcium alginate-based wound dressings are commercially available. After application of the calcium alginate-based wound dressings on wounds, sodium from the wound’s exudates and calcium from alginate dressings will undergo ion exchange, forming a soluble sodium alginate gel. Alginate was thus selected to formulate the digitoxigenin formulation and olive oil was a vehicle to dissolve digitoxigenin.

The objective of this study was to evaluate the possibility of digitoxigenin and its topical formulation for wound healing effect. We first examined if digitoxigenin could stimulate collagen synthesis in human dermal fibroblasts and exert the wound healing activity in a rat excisional wound model. Next, the microspheres containing both digitoxigenin and olive oil were prepared using alginate and the microsphere-dispersed gel formulation was investigated for in vitro drug release and in vivo wound healing effect.

## 2. Materials and Methods

### 2.1. Materials

Digitoxigenin and olive oil (highly refined, low acidity) were purchased from Sigma-Aldrich (St. Louis, MO, USA). Alginic acid sodium salt (low viscosity) was from MP Biomedicals (Solon, OH, USA). Tween 85 (Acros Organics, Morris, NJ, USA) and Span 85 (Sigma-Aldrich, St. Louis, MO, USA) were used in the preparation of digitoxigenin formulations. Other reagents used were isooctane (Acros Organics, Morris, NJ, USA) and calcium chloride dehydrate (Fisher Scientific, Springfield, NJ, USA). Phosphate buffered saline (PBS) of pH 7.4 was prepared by dissolving PBS tablets (MP Biomedicals, Solon, OH, USA) in deionized water, and sodium azide (Acros Organics, Morris, NJ, USA) was added at a concentration of 0.02% (*w*/*v*) as a bacteriostat. High performance liquid chromatography (HPLC) grade methanol, water, and formic acid were purchased from Fisher Scientific (Pittsburgh, PA, USA).

### 2.2. Animals

Sprague-Dawley rats (male and female, 240–260 g) were purchased from Hilltop Lab Animals (Scottdale, PA, USA). All of the experiments were conducted under the approval of the Institutional Animal Care and Use Committee at Marshall University.

### 2.3. Stimulation of Collagen Synthesis in Human Dermal Fibroblast Cells

In vitro stimulation of collagen synthesis activity of digitoxigenin was investigated in human dermal fibroblast (HDF) cells (product No. CCD-1072Sk, ATCC No: CRL-2088) supplied from ATCC (Manassas, VA, USA). HDF cells were cultured with ISCOVE’S modified Dulbecco’s medium (Sigma-Aldrich, St. Louis, MO, USA) containing 10% fetal bovine serum (FBS, Atlanta Biologicals, Flowery Branch, GA, USA). After the HDF cells grew to confluence, the medium was removed and the cells were incubated with the medium containing 1% FBS for 12 h to avoid any possible interference due to growth factors present in the serum. The cells were then treated with the medium (negative control, containing 10% FBS but no digitoxigenin or ouabain), ouabain (positive control, 1 nM dissolved in the medium containing 10% FBS), or digitoxigenin (0.1, 1 or 10 nM dissolved in the medium containing 10% FBS). After treatment for 24 h, cell lysates were prepared by washing the cells twice with ice-cold PBS, followed by incubating them on ice for 20 min with a lysis buffer containing 50 mM HEPES sodium, 50 mM NaCl, 10% glycerol, 1% Nonidet P-40, 0.25% Na-Dexycholate, and protease and phosphatase inhibitors. Cell lysates were collected and used immediately or stored at −80 °C. 

Western blot analysis was performed as described previously [6]. Briefly, cell lysates were dissolved in loading buffer and proteins (10 μg/lane) were separated by SDS-PAGE using 8% Tris·HCl ready precast gels (Bio-Rad, Hercules, CA, USA). After separation, proteins were transferred onto polyvinylidene difluoride (PVDF) membranes. The membranes were blocked with 5% non-fat dry milk (Bio-Rad, Hercules, CA, USA) in Tris-buffered saline that was supplemented with 0.05% Tween 20 (TBS-T) at room temperature for 1 h and then incubated with primary antibody (Goat-Anti-Type 1 Collagen-UNLB, Southern Biotech, Birmingham, UK) in blocking buffer at 4 °C overnight. After being washed in TBS-T, the membranes were incubated with secondary antibody in the blocking buffer for 2 h at room temperature. After being washed in TBS, the membranes were developed with ECL (Amersham Biosciences, Piscataway, NJ, USA). For loading controls, tubulin was probed. The images captured on X-ray film were scanned and quantified by using Image J software.

### 2.4. Preparation of Digitoxigenin Microspheres

Digitoxigenin-loaded alginate microspheres were prepared by an emulsification technique with modifications from the literatures [10,11]. Briefly, 250 μL 150 μg/mL digitoxigenin in olive oil were dispersed in 5 g 2% *w*/*w* alginate solution using a homogenizer (Polytron PT 2100; Kinematica, Lucern, Switzerland) at 11,000 rpm for 1 min. After the addition of 7.5 g isooctane containing 0.254 g Span 85 into the alginate solution followed by homogenization for 1 min, 1 g aqueous solution containing 0.136 g Tween 85 was added and homogenized for 1 min. Two grams of 25% calcium chloride were added and the mixture was homogenized for 10 min to allow for the calcium to react with the sodium alginate. Isooctane was decanted after centrifugation at 4400 rpm for 5 min and microspheres were collected. The microspheres were washed with 2 mL deionized water three times and dried at room temperature for the in vitro and in vivo studies. 

### 2.5. Digitoxigenin Content Assay

The amounts of digitoxigenin loaded to the microspheres were determined by extracting digitoxigenin from the microspheres and subsequent assaying by LC-MS/MS. Microspheres of 0.03 g were dispersed in 1 mL PBS and then sonicated in an ultrasonic bath for 1 h. The mixture was centrifuged at 10,000 rpm for 5 min and the supernatant was collected. The residue was subjected to two more extractions and the supernatants were collected. Digitoxigenin was extracted from the supernatant with methanol and analyzed by LC-MS/MS. The amount of digitoxigenin in the microspheres was determined.

### 2.6. In Vitro Release Study 

Digitoxigenin released from the microsphere was determined using Franz diffusion cells with an effective diffusion area of 0.64 cm^2^ and a MF-Millipore membrane (0.45 μm) that was sandwiched between the donor and receptor compartments. The digitoxigenin-loaded microspheres of 0.05 g were dispersed in 600 μL 2% (*w*/*w*) alginate solution containing 0.9% (*w*/*v*) NaCl and then loaded to the donor chamber. The receptor solution was 5 mL PBS containing 0.02% (*w*/*v*) sodium azide and was kept at 37 °C ± 1 °C. One milliliter of the receptor solution was collected at 20 and 40 min as well as at 1, 1.5, 3, 3.5, 4, and 7 h, with replacement of a fresh receptor solution to maintain the volume of the receptor solution constant. The samples were assayed for digitoxigenin by LC–MS/MS. The amounts of digitoxigenin released from the microspheres were determined and plotted versus the time.

### 2.7. Wound Healing Study

The rats were randomly divided into seven groups and seven rats for each group. The rats were anesthetized with intraperitoneal injection of ketamine/xylazine (30–50 mg/kg & 5–10 mg/kg, respectively). Prior to wound creation, buprenorphine was administered intraperitoneally (0.01–0.05 mg/kg). The hair in an area of about 500 mm^2^ in the dorsal skin of the rat was shaved and the hair-removed area was wiped with alcohol swab (Becton-Dickinson, Franklin Lakes, NJ, USA). Full-thickness skin biopsy was performed with an 8.0-mm sterile punch biopsy (Acu-punch, Acuderm, Fort Lauderdale, FL, USA). Two symmetric lesions with at least 15 mm apart were made in each rat and received two different treatments immediately after wound creation; one wound was treated with a vehicle and the other with a digitoxigenin-loaded formulation. The seven treatment groups were (1) untreated (no vehicle or digitoxigenin-loaded formulation applied), (2) olive oil, (3) 2% *w*/*w* alginate solution containing 0.9% sodium chloride, (4) digitoxigenin in olive oil (1 ng/mL), (5) digitoxigenin in olive oil (0.1 ng/mL), (6) digitoxigenin in 2% alginate solution (1 ng/mL), and (7) digitoxigenin-loaded microspheres in 2% alginate solution containing 0.9% sodium chloride. The digitoxigenin microsphere gels were freshly prepared by dispersing 10 mg microspheres in 1 mL 2% *w*/*w* alginate solution containing 0.9% NaCl.

The vehicle or the digitoxigenin-loaded formulations of 100 µL was first applied to a Curad clear water proof bandage (Medline Industries, Mundelein, IL, USA), which was cut into two rectangle pieces (1.25 × 0.75 cm). The wounds were immediately covered with the Curad bandages after wound creation and an adhesive porous tape (Kendall, Covidien, Mansfield, MA, USA) was applied to hold the Curad bandage in place. To ensure the bandages were not removed by rats and wound contraction was inhibited in the wound healing process, additional self-adhesive wrap (Coban, 3M Health Care, St. Paul, MN, USA) and adhesive tapes were applied. At 3, 7 and 10 days following the wounding, the bandages were gently removed and digital photographs were taken immediately. A new bandage was applied to the wounds, but no vehicle or digitoxigenin was added. Photographs were analyzed with Image J and the percentage of wound open was calculated as the remaining wound area with respect to the initial wound area.

### 2.8. LC–MS/MS Assay

The LC–MS/MS assay method was developed and modified from the literature [8]. The LC–MS/MS system consisted of an Agilent 1290 series UHPLC system and an Agilent 6490 Triple Quadrupole (QqQ) mass spectrometer equipped with Agilent Jet-Stream ESI interface. Chromatographic separation was carried out on an Agilent Poroshell 120 EC-C18 column (2.1 × 50 mm I.D., 2.7 μm) with an Agilent UHPLC guard cartridge (2.1 × 5 mm I.D., 2.7 μm) at room temperature with a flow rate of 200 μL/min. The mobile phase consisted of water (0.1% formic acid)–methanol (0.1% formic acid) (40:60, *v*/*v*), and the total run time was 4.0 min. ESI source was operated in the positive ionization mode. The autosampler temperature was set at 10 °C. The general source settings were as follows: gas temperature 220 °C; gas flow 12 L/min; nebulizer 22 psi; sheath gas temperature 250 °C; sheath gas flow 12 L/min; capillary voltage 3500 V; and, nozzle voltage 1500 V. The fragmentor voltage was 380 V for all mass transitions, and both scanning quadrupoles (Q1 and Q3) were set to unit resolution. Selective reaction monitoring (SRM) for digitoxigenin was conducted by monitoring the precursor-product ion transition of *m*/*z* 375.5 → 339 and the collision energy was 30 eV. LC–MS/MS data acquisition and processing were performed using Agilent’s MassHunter Quantitative and Qualitative Analysis software (version B.06.00). The calibration curves were linear over the concentration range of 0.1–20 ng/mL.

### 2.9. Statistical Analysis

All of the data are presented as mean ± standard error (SE). Statistical differences of means were determined using one-way analysis of variance (ANOVA) and are considered to be significant at a level of *p* < 0.05.

## 3. Results

### 3.1. Stimulation of Collagen Synthesis in Human Dermal Fibroblast (HDF) Cells

**Figure 1:**
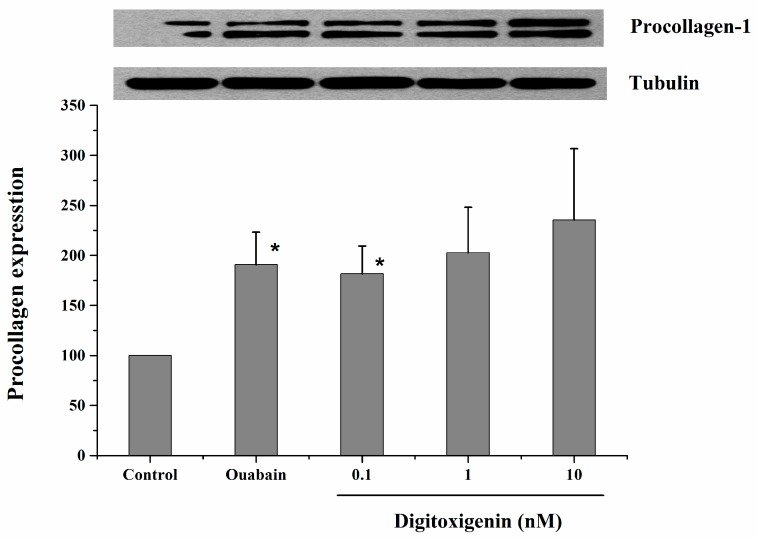
Western blot analysis of procollagen synthesized by HDFs exposed to 3 different concentrations of digitoxigenin. The data are presented as mean ± standard error (*n* = 4). *, *p* < 0.05 versus control.

Figure 1 shows the western blot analysis of procollagen synthesized by HDFs following exposure to three different concentrations of digitoxigenin. After the HDFs were exposed to digitoxigenin at 0.1 nM, the expression of procollagen was significantly increased (*p* < 0.05). For the HDFs that were treated with digitoxigenin at 1 nM and 10 nM, the expression of procollagen was increased but the difference was insignificant (*p* > 0.05), which might be related to the large data variability and small sample size. No significant dose-dependence for digitoxigenin was observed.

### 3.2. In Vitro Release Study

**Figure 2:**
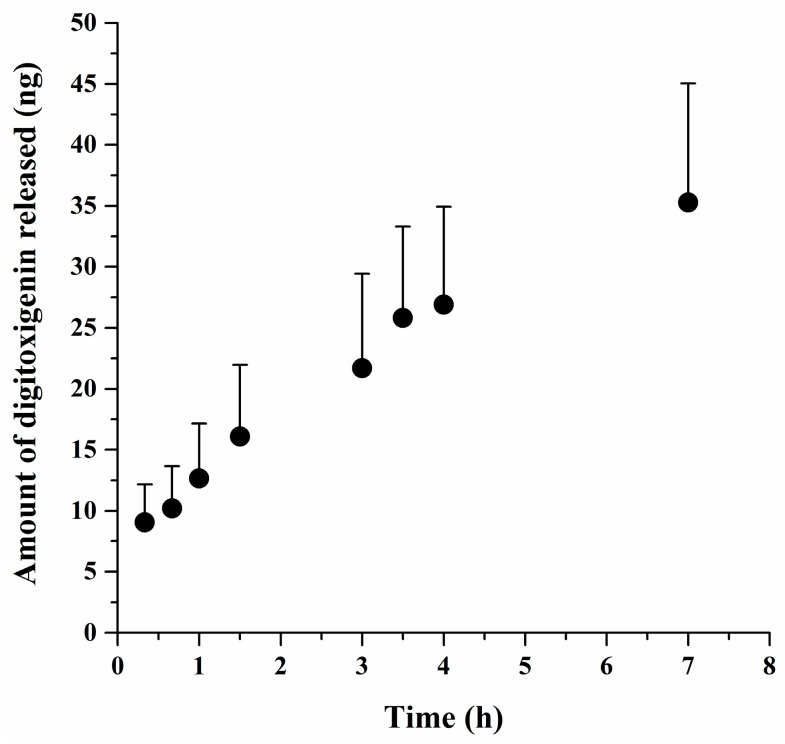
The amounts of digitoxigenin released from alginate-digitoxigenin microspheres versus time. The data are presented as mean ± standard error (*n* = 4).

Figure 2 presents the amounts of digitoxigenin released from the microspheres over time. Digitoxigenin was gradually released from the microspheres. At 7 h, about 35 ng or 60% of digitoxigenin was released. These results implied that the microspheres could be used as a delivery system for digitoxigenin. The average amount of digitoxigenin in the microspheres was approximated as 1153 ± 81 ng/g microspheres (mean ± SE, *n* = 6). It was thus estimated that the dose of digitoxigenin applied in the wound healing study was 1.2 ng.

### 3.3. Wound Healing Study

**Figure 3:**
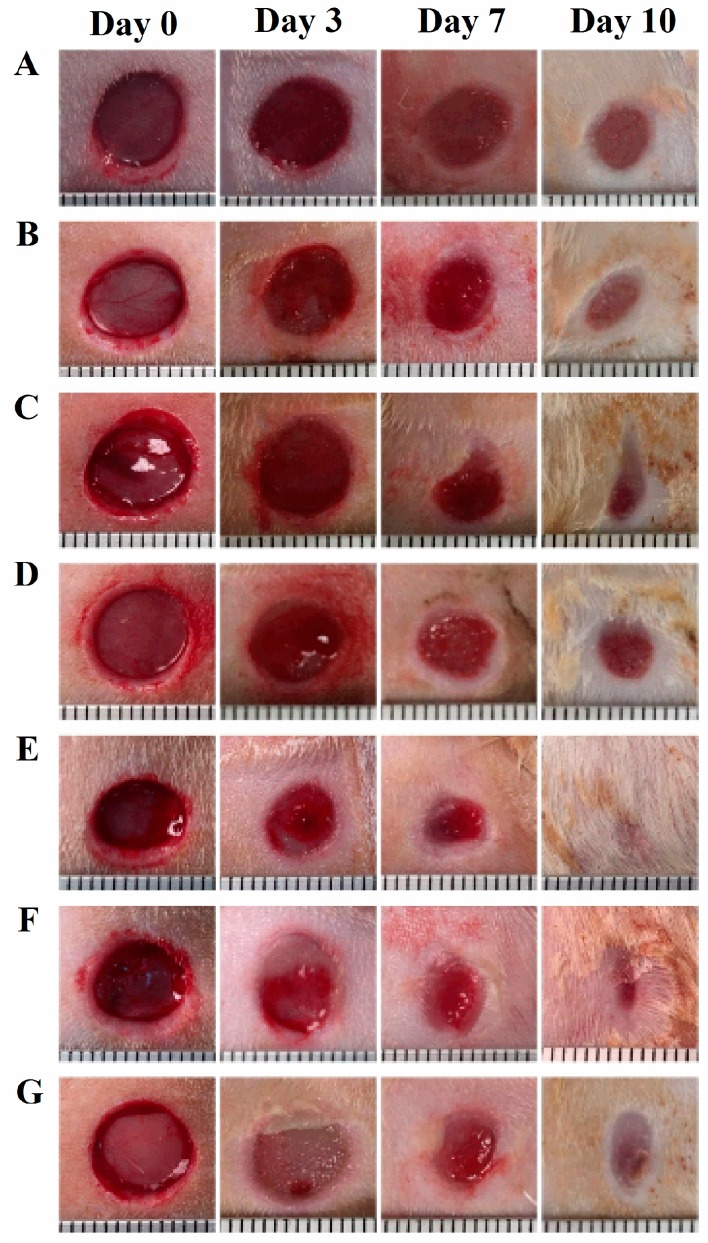
Representative images of wounds following the treatments of (**A**) untreated, (**B**) olive oil, (**C**) 2% alginate solution, (**D**) digitoxigenin in olive oil at a dose of 0.1 ng, (**E**) digitoxigenin in olive oil at a dose of 0.01 ng, (**F**) digitoxigenin in 2% alginate solution at a dose of 0.1 ng, and (**G**) digitoxigenin microspheres at a dose of 1.2 ng. The wounds received digitoxigenin treatments for three days after wounding.

**Figure 4:**
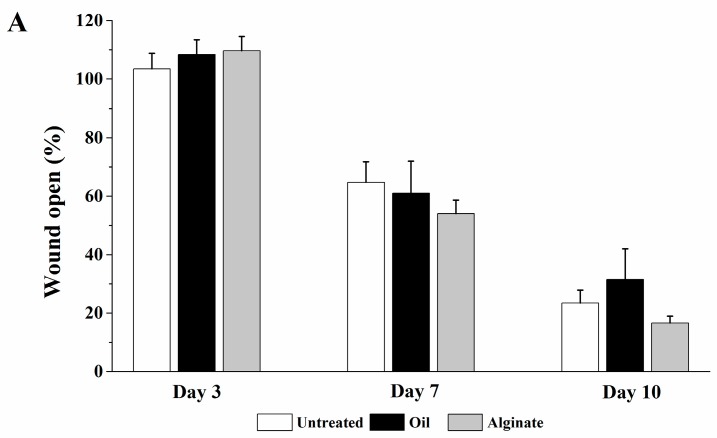

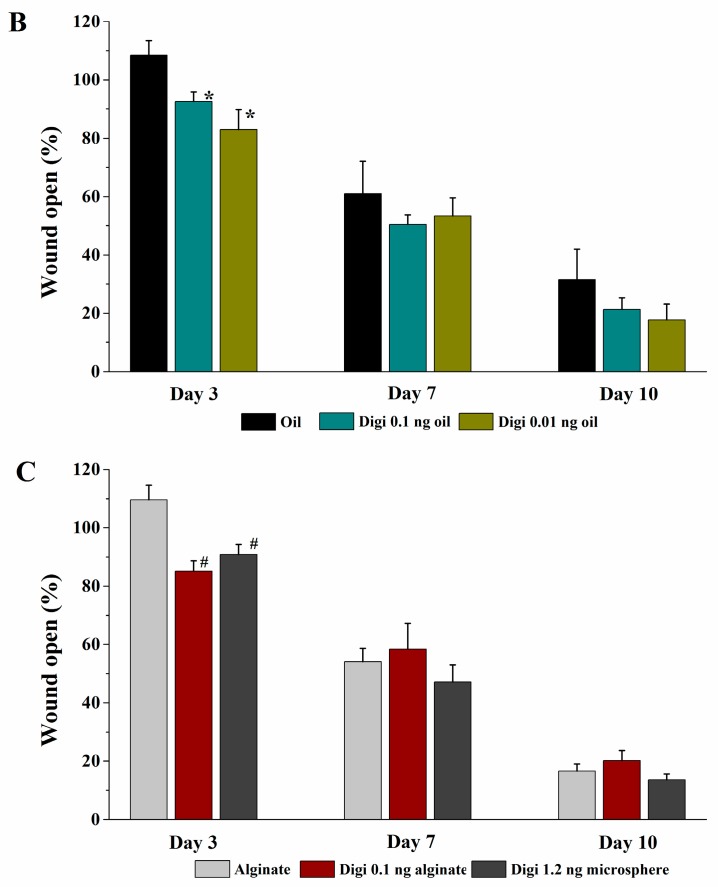
Percentages of wound open at days 3, 7, and 10 after wounding. The wounds received the digitoxigenin treatments for 3 days after wounding. The data are presented as mean ± standard error (*n* = 7). (**A**) Untreated group (Untreated) and groups treated with olive oil (Oil) and 2% alginate solution (Alginate); (**B**) Groups treated with digitoxigenin 0.1 ng (Digi 0.1 ng oil) and 0.01 ng (Digi 0.01 ng oil) in olive oil compared with the one treated with olive oil (Oil); * *p* < 0.05 versus the olive oil group; (**C**) Groups treated with digitoxigenin 0.1 ng (Digi 0.1 ng alginate) and digitoxigenin microspheres in alginate solution at a dose of 1.2 ng (Digi 1.2 ng microsphere) when compared with the one treated with alginate solution (Alginate); # *p* < 0.05 versus the sodium alginate group.

Representative images of wounds following different treatments are presented in Figure 3 and the percentages of wound open at days 3, 7, and 10 after wounding are shown in Figure 4. When compared with the untreated group, no significant difference in wound open at days 3, 7, and 10 was observed for blank olive oil and blank alginate groups (Figure 4A). While olive oil and alginate were reported to possess wound healing activity in some cases [12,13], this effect was not shown in our study with the single dose application method. As shown in Figure 4B, digitoxigenin in olive oil solution at the doses of 0.01 ng and 0.1 ng significantly decreased the wound open at day 3 post wounding (*p* < 0.05) as compared with blank olive oil. This indicated that digitoxigenin could be delivered from the olive oil solution and exert wound healing activity. Figure 4C shows that digitoxigenin in alginate solution significantly decreased the wound open at day 3 with respect to the alginate control group (*p* < 0.05); this further implied the wound healing effect of digitoxigenin. Similarly, as shown in Figure 4C, digitoxigenin microspheres in alginate solution increased the wound healing at day 3 after wound creation compared with blank alginate solution (*p* < 0.05). These results suggested that alginate could be used in the formulation of digitoxigenin and digitoxigenin was released from the alginate microspheres to elicit wound healing effect. When digitoxigenin was not applied, no significant differences in the wound healing activity were observed at days 7 and 10 post wounding for the digitoxigenin treatment groups as compared with their respective oil or alginate control group (*p* > 0.05). Additionally, there were no statistical differences between the different doses applied in this study (*p* > 0.05).

## 4. Discussion

### 4.1. Stimulation of Collagen Synthesis by Digitoxigenin

It is accepted that Na/K-ATPase has an ion-pumping independent receptor function through its direct interaction with Src kinase to form a functional receptor complex, which can be stimulated by its ligands, such as CTSs [14]. Binding of CTSs with the Na/K-ATPase/Src complex receptor was found to increase collagen synthesis via the PLC/PKC signal transduction [6]. As collagen deposition is one of the most important mechanisms in a wound healing process, it was suggested that CTSs could have wound healing activities by the stimulation of collagen synthesis. In the present study, we first examined if digitoxigenin can stimulate collagen synthesis using an in vitro cell culture model.

Our in vitro cell culture study (Figure 1) has shown the significantly increased expression of procollagen in the HDFs treated with digitoxigenin at 0.1 nM (*p* < 0.05). This result is consistent with the prior finding that CTS compounds digoxin and marinobufagenin (MBG) stimulated collagen synthesis in fibroblasts [4,6]. Despite the increased expression of procollagen at higher concentrations of digitoxigenin (1 and 10 nM), no statistical differences between treated groups and the control group were observed. Unlike MBG in the previous study [6], no dose-dependent increase in the expression of procollagen was found for digitoxigenin. This might be related to their different binding properties. With increasing the concentration of digitoxigenin applied, the receptor binding sites can become saturated, and therefore, the response will not increase with dose. While it is not known if the IC_50_ for the Na/K-ATPase pump inhibition can reflect the binding capability of CTS with the Na/K-ATPase as the receptor and at which concentration the receptor binding sites will be saturated with digitoxigenin, the lower IC_50_ value for digitoxigenin (0.22 µM) than for digoxin (1.76 µM) (unpublished data) might suggest that the saturation would occur at a lower concentration of digitoxigenin. The unobserved dose-dependent response might be also related to the large data variability and the possible involvement of other signal transductions to stimulate collagen synthesis in HDF following exposure to digitoxigenin [15].

### 4.2. Wound Healing Effect of Digitoxigenin

In the previous study with digoxin [4], olive oil was found necessary for the successful delivery of digoxin for wound healing. In our animal study, olive oil was thus also used as a vehicle for digitoxigenin and its formulation. In addition, alginate was selected for topical formulation of digitoxigenin due to its desirable properties for wound healing [16]. To examine the feasibility of using digitoxigenin for wound healing, we first performed the in vivo wound healing studies using digitoxigenin in olive oil or alginate solution. Figure 3 and Figure 4 show that digitoxigenin at the doses of 0.01 and 0.1 ng in olive oil significantly decreased the wound open when compared with the olive oil control group and the wound healing was promoted with the digitoxigenin treatment for 3 days. Digitoxigenin in alginate solution at the dose of 0.1 ng also accelerated the wound healing as evidenced by the decreased wound open three days after wounding as compared with the alginate control group. After removal of digitoxigenin from the wound site at day 3 after wounding, the wound healing effect of digitoxigenin was not maintained. These results suggest that digitoxigenin increased the wound healing when it was applied. The finding in this study is consistent with the results in the previous study where digoxin was dosed over nine days post wounding [4]. Repeated dosing is needed to exert the wound healing effect for a prolonged period of time and warrants further study.

As a topical formulation with the capability of loading both olive oil and digitoxigenin is desired for topical administration, the digitoxigenin microspheres dispersed in an alginate gel were thus formulated and tested for wound healing in vivo. It can be seen from Figure 3 and Figure 4 that digitoxigenin-loaded microsphere gels also exerted wound healing effect at day 3 after wounding. The accelerated wound healing was not observed at days 7 and 10 when no digitoxigenin formulation was applied. These results suggest that digitoxigenin was released from the microspheres in vivo to elicit the wound healing effect. The alginate microsphere-based gel might be a potential platform for topical formulation of digitoxigenin. The formulation might be further manipulated to prolong the wound healing effect.

### 4.3. Feasibility and Potential of Using Digitoxigenin for Wound Healing

The results from the present study suggest that digitoxigenin exhibited the wound healing activity at a dose as low as 0.01 ng. This is in contrast with the dose of digoxin (2.3 ng) that was used for wound healing in the previous study [4]. Such a low effective dose would be highly desirable for clinical application of digitoxigenin. It is well known that systemic administration of CTS compounds, like digoxin, increases the risk of cardiac toxicity, presenting a serious problem that requires close therapeutic dose monitoring. The cardiac toxicity is attributed to the ion pumping function of the Na/K-ATPase that is related to the levels of CTSs in the systemic circulation [17]. Topical application of digitoxigenin for wound healing could allow for using low doses at the wound site while minimizing systemic absorption of digitoxigenin and thus preventing systemic side effects. Moreover, it is also known that the concentrations of CTS needed to activate the receptor function of the Na/K-ATPase are much lower than those that are needed to inhibit the pump function of the Na/K-ATPase [18]. It can be expected that the effective doses of digitoxigenin for wound healing may be too low to inhibit the pump function of the Na/K-ATPase, and subsequently to result in the pump-related cardiac toxicity. Many CTSs have been clinically used at the concentrations far beyond what was used in the topical administration [19]. To this end, digitoxigenin might be a potential therapeutic for wound healing and the cardiac toxicity would not be a major concern.

While this study has demonstrated the feasibility of using topical digitoxigenin for wound healing, there were limitations in the study. First, histological and biochemical assays were not performed in the present study to examine the roles of digitoxigenin in wound healing. Increased collagen deposition and wound healing should be confirmed in vivo by analyzing wound tissues. Our unpublished data from a separate research using digitoxigenin and a wound model of rats suggested the effect of digitoxigenin on collagen deposition in vivo (data not shown). Vascularization and other mechanisms may be involved in the wound healing and should be investigated in future studies to reveal the underlying mechanisms of digitoxigenin in wound healing. Second, single dosing regimen was used in our present study and the wound healing effect was only observed when digitoxigenin was applied. It would be impossible to evaluate the overall wound healing outcomes following the single dose of topical digitoxigenin and to elucidate the mechanisms of action, given the complex wound healing process with several overlapped phases. Repeated doses over a long period of time would be needed to confirm the efficacy of digitoxigenin. Third, while we had taken special procedures to minimize wound contraction in rats, it would be worthwhile to further determine the effect of digitoxigenin on wound healing in animal models better resembling the wound healing process in humans. To sum up, the present study implies the feasibility of digitoxigenin and its topical formulation for wound healing. However, digitoxigenin as a promising wound healing candidate warrants future studies to validate the efficacy and better understand the mechanisms. This was the first investigation of using digitoxigenin in wound healing, and would present a new direction to explore novel therapeutics for wound healing. 

## 5. Conclusions

This was a pilot feasibility study to determine (1) if digitoxigenin, a cardiac aglycone, had effects on collagen synthesis in vitro and wound closure in vivo as previously reported for digoxin, a cardiac glycoside; and, (2) if a formulation could be developed for topical application. The results showed that digitoxigenin increased collagen synthesis in human dermal fibroblasts and decreased wound open in a rat excisional wound model as compared with the vehicle controls. The digitoxigenin-loaded microsphere gel exerted the wound healing effect similar to digitoxigenin in vehicles and could serve as a convenient dosage form for topical application. However, topical digitoxigenin need further investigation to prove efficacy and understand underlying mechanisms. While this single dosing study could provide an initial understanding of the effect of digitoxigenin in the early phase of wound healing, multiple dosing studies will be necessary to understand the effect of digitoxigenin in the later phase of a wound healing process. The results from this study imply that digitoxigenin might be a promising candidate for future development to be a wound healing therapeutics.
